# Early mobilization in patients with aneurysmal subarachnoid hemorrhage: a prospective observational study

**DOI:** 10.1093/ptj/pzag031

**Published:** 2026-03-27

**Authors:** Sabrina Hernandez, Claire Tipping, Adam M Deane, Michael Wei, Wendy Bower, Alexios Adamides, Anais Charles-Nelson, Peter Thomas, Jonathan Tomkins, Jane Larkin, Carol L Hodgson

**Affiliations:** Allied Health Department, The Royal Melbourne Hospital, Parkville, VIC 3050, Australia; Australian and New Zealand Intensive Care-Research Center, School of Public Health and Preventive Medicine, Monash University, Parkville, VIC 3050, Australia; Physiotherapy Department, The Alfred Hospital, Melbourne, VIC 3004, Australia; Department of Intensive Care, The Royal Melbourne Hospital, Parkville, VIC 3050, Australia; Department of Neurosurgery, The Royal Melbourne Hospital, Parkville, VIC 3050, Australia; Allied Health Department, The Royal Melbourne Hospital, Parkville, VIC 3050, Australia; Faculty of Medicine, Dentistry and Health Science, University of Melbourne, Melbourne, VIC 3010, Australia; Department of Neurosurgery, The Royal Melbourne Hospital, Parkville, VIC 3050, Australia; Australian and New Zealand Intensive Care-Research Center, School of Public Health and Preventive Medicine, Monash University, Parkville, VIC 3050, Australia; The Royal Brisbane and Women’s Hospital, Herston, QLD 4029, Australia; Allied Health Department, The Royal Melbourne Hospital, Parkville, VIC 3050, Australia; Allied Health Department, The Royal Melbourne Hospital, Parkville, VIC 3050, Australia; Australian and New Zealand Intensive Care-Research Center, School of Public Health and Preventive Medicine, Monash University, Parkville, VIC 3050, Australia; Physiotherapy Department, The Alfred Hospital, Melbourne, VIC 3004, Australia

**Keywords:** Early mobilization, aneurysmal subarachnoid haemorrhage, mobilization barriers, independent walking

## Abstract

**Importance:**

Patients with aneurysmal subarachnoid hemorrhage (aSAH) represent a cohort with limited evidence to guide mobilization practices.

**Objective:**

The objective was to describe acute mobilization practices, outcomes, and barriers to mobilization in patients following aSAH.

**Design:**

The design of the study was a single-center prospective, observational study.

**Setting:**

This study was conducted in the acute ward and intensive care unit of a tertiary neurosurgical referral center.

**Participants:**

Participants were adult (≥18 years) patients post-aSAH with secured aneurysms.

**Exposure:**

Mobilization practices were delivered during physical therapist sessions up to 14 days post-aneurysm repair.

**Main Outcomes and Measures:**

Severity was classified using the World Federation of Neurological Surgeons scale, dichotomizing into “good” (Grade I to II) and “poor” (Grade III to V) clinical status. Mobilization outcomes were measured using the Mobility Scale for Acute Stroke (MSAS), with independent walking assessed.

**Results:**

A total of 102 patients participated with 69 (67.6%) classified as “good” grade and 90 (88.2%) of patients mobilized within the first 14 days. Data were collected from 603 planned mobilization sessions, with barriers to mobilization encountered in 193 (32.0%) of these sessions, primarily due to neurological instability (*n* = 80, 41.5%) and hemodynamic instability (*n* = 43, 22.3%). Overall, the highest median MSAS score achieved was 32 (IQR = 10 to 36). By 2 weeks, 65.2% of patients with a “good” clinical status walked independently versus 12.9% in the “poor” group.

**Conclusions:**

While most patients mobilized, physiological instability commonly prevented mobilization activities. Independent walking by 2 weeks was significantly more common in patients with “good” clinical status. These findings underline the importance of careful screening and monitoring during mobilization in the acute period.

**Relevance:**

This study underscores the need for further research into optimal mobilization strategies for improving outcomes in patients with aSAH.

## Introduction

Aneurysmal subarachnoid hemorrhage (aSAH) is a form of stroke with high mortality and morbidity rates; approximately one-third of patients die in hospital^1^ and around half of all patients experience long-term disability with permanent physical, psychological, or cognitive impairments.^2^^,^^3^ aSAH typically affects adults aged 45 to 64 years.^4^ As many survivors are unable to return to work,^5^ this neurological disorder generates a significant socioeconomic burden.

Due to the systemic complications that can occur as direct result of the bleed into the subarachnoid space, patients experience a prolonged and complicated recovery. In approximately one-third of patients delayed cerebral ischemia provokes neurological deterioration.^6^^,^^7^ Vasospasm, the radiological narrowing of cerebral arteries, occurs in two-thirds of patients post-aSAH.^6^ Both vasospasm and delayed cerebral ischemia emerge within the first 14 days post-bleed and are associated with cerebral infarction and poor functional outcomes.^8^ The association between physical activities and/or changes in position and functional recovery during this high-risk period is not well understood.

Following acute brain injury, early mobilization may enhance neuroplasticity and functional recovery during the initial phase post injury.^9^^,^^10^ Earlier mobilization may counteract the ill-effects of bedrest such as respiratory infections, pulmonary embolism and pressure injuries. Despite specific focus on early rehabilitation after stroke, mobilization practices in the acute phase following aSAH are poorly described. Current clinical practice guidelines for the mobilization of patients after aSAH are based on low quality evidence^8^^,^^11^ and provide limited guidance on timing, frequency and type of mobilization activities that should be delivered in the acute and intensive care settings, or the optimal levels of mobilization that should be achieved while in hospital. Furthermore, the influence of potential barriers (such as patient, communication and organizational-related factors) and safety concerns (eg, hemodynamic or neurological instability) prior to or during mobilization activities has been inadequately studied.

Given the lack of evidence, it is critical to further define standard care for patients with aSAH, including timing and type of mobilization activities delivered, the barriers that prevent mobilization, and the frequency of mobilization-related safety events. Hence, this study aimed to describe in patients with aSAH (1) current mobilization practices; (2) barriers to mobilization; (3) safety events associated with mobilization during the first 14 days post-repair; and (4) the association between level of mobilization in patients with a good clinical status on admission compared to those with a poor clinical status. The findings of this study will be used to inform interventional research examining the efficacy of early mobilization in this patient population.

## Methods

### Study design

This prospective, single-site, observational study of usual physical therapy practice was conducted between April 2022 and October 2023. Ethical approval, including a waiver of consent was obtained from the institution’s Human Research Ethics Committee (Number: HREC/81210/MH-2021) for use of routinely collected hospital data and to align with the National Statement on Ethical Conduct in Human Research (Chapter 4.5) given many patients following aSAH may have had limited capacity early following admission.^12^ The Strengthening of reporting of observational studies in epidemiology guideline was followed.

### Setting and timeline

Patients admitted following aSAH were recruited from the Intensive Care Unit (ICU) or neurosurgical ward of a large public tertiary hospital. The neurosurgical ward includes a high dependency unit (HDU) where a higher level of observation or nursing care can be provided while patients remain under the care of the neurosurgical team. However, all patients who require organ support, such as invasive mechanical ventilation or vasoactive drugs, or continuous intra-arterial blood pressure monitoring, are cared for by the ICU team in a “closed” area. Patients in this setting generally undergo aneurysm repair within 24 to 48 hours. An external ventricular drain (EVD) may be inserted to treat hydrocephalus. From day 1 following repair of the culprit aneurysm physical therapists typically screen patients for suitability to participate in mobilization; this occurs daily in ICU, weekdays on the neurosurgical wards, and on weekends if the surgical clipping had been completed the preceding day. Patients remained in hospital for close monitoring for the emergence of neuroradiological complications for approximately 2 weeks following aSAH. Treatment interventions for delayed cerebral ischemia and vasospasm included intravenous infusion of vasopressors, intra-arterial balloon angioplasty or intra-arterial vasodilators to reduce the risk of further neurological injury.

### Participants

Patients were prospectively screened from neurosurgical unit admission lists accessed via electronic medical records. Eligible patients included adults, aged 18 years or older, admitted to the neurosurgical ward or the ICU with confirmed aSAH (diagnosed on computed tomography (CT) or xanthochromia on lumbar puncture), who had undergone repair of their ruptured aneurysm either by surgical clipping or endovascular coiling. The confirmation of diagnosis of aSAH was made by neurosurgeon or intensivist and documented in the electronic medical record. Patients were ineligible if the subarachnoid hemorrhage was due to causes other than aneurysm rupture (eg, trauma, arteriovenous malformation-related, or perimesencephalic), they were unable to walk independently in the month prior to admission (with or without a gait aid), had prior cognitive impairment (eg, intellectual disability or dementia), previous history of neurological disease or insult with neurological impairment, any concurrent traumatic injuries that impaired mobility and patients in whom death was imminent or inevitable, as recorded in the electronic medical records.

### Clinical and hospital variables data collection

Study researchers collected demographic data from the electronic medical records for all enrolled patients which included age, sex, co-morbidities, and pre-admission level of function using the modified Rankin Scale,^13^ location of ruptured aneurysm, time from ictus to aneurysm repair of the culprit aneurysm, need for ICU admission, ICU length of stay, and hospital length of stay. Where the ictus time was unknown, onset was defaulted to midday of the reported day of symptom onset. Mortality during hospital admission and discharge disposition for those who survived were also recorded.

Routinely collected data were extracted from electronic medical records. Treatment variables collected included mode of treatment of the ruptured aneurysm (surgical clipping or endovascular coiling), treatment for delayed cerebral ischemia (intravenous vasopressors, intraarterial vasodilators, or balloon angioplasty), insertion of an EVD and tracheostomy or mechanical ventilation during their acute hospital admission. The World Federation of Neurological Surgeons (WFNS) scale data was also collected in order to describe the severity of the aSAH at the time of hospital admission.^14^ WFNS scale categories range from grade 1 (lowest clinical severity) to 5 (worst clinical severity) and is a valid clinical prognostics measure, with the presence of hemiparesis and/or aphasia and level of consciousness being important predictors of disability and mortality respectively.^14^^,^^15^ For the purpose of this study, and consistent with previous research in early mobilization, patients were classified into groups on admission based on clinical status: “good” (WFNS 1–2) indicating no neurological deficit and consciousness largely intact and “poor” (WFNS 3–5) indicating focal neurological deficit and/or significantly impaired consciousness.^16^^,^^17^ To determine the risk of cerebral vasospasm the modified Fisher Scale score was collected. This is graded using scores 0 to 4 based on the extent of SAH and intraventricular hemorrhage, with a higher score indicating a greater volume of hemorrhage.^18^ The neuroradiological complications of cerebral infarct, radiological vasospasm and delayed cerebral ischemia were collected from the electronic medical records. Cerebral infarct was defined as new infarcts not evident on admission scan and in patients found to have radiologic vasospasm. Radiologic vasospasm and delayed cerebral ischemia were defined as per the Neurocritical Care Society Guidelines for aSAH management.^19^ To measure immobility-related complications, incidence of pulmonary embolism and deep vein thrombosis were recorded for the duration of the patient’s hospital admission.

### Outcome measures

For each routine physical therapist session completed and for those that didn’t occur due to at least 1 reported barrier, the treating physical therapist recorded any mobilization episode as part of their usual documentation, retained within the patient’s electronic medical records. These data were extracted by a study researcher from the electronic medical records and are outlined in the mobility case report form (Suppl. Material 1). Data collection commenced from the first day following the repair of the ruptured aneurysm up to day 14 post-aneurysm repair, death or discharge from hospital (whichever occurred sooner). Data were later extracted from the electronic medical records (Epic Systems, Verona, WI, USA)^20^ by a study researcher.

The mobility level achieved each physical therapist session and the highest level of mobilization achieved during the first 14 days post-aneurysm repair, was measured using the Mobility Scale for Acute Stroke (MSAS).^21^ To reflect task-specific mobilization activities and in line with commonly used metrics in early stroke rehabilitation, timing and proportion of patients who achieved independent out-of-bed mobilization milestones of sitting, standing and walking were used.^22^^,^^23^ These were defined as achieving score of 2 or greater on the MSAS item 2 (lie to sit), MSAS item 4 (sit to stand) and MSAS item 6 (walking 10 m with or without a gait aid) respectively. Patients that did not mobilize in the first 14 days post-aneurysm repair were scored a total score of 6 indicating unable (score of 1) for all 6 mobility items. If a patient was discharged from physical therapist services within the 14-day data collection period, the day 14 MSAS scores were completed on day of discharge, based on the last mobilization session completed. Physical therapists were given a 24-to-48-hour window before or after day 14 to measure the MSAS, to accommodate staffing constraints and the absence of routine mobility reviews on weekends.

To further describe mobilization practices during physical therapist sessions, key variables were recorded including total number of mobilization sessions completed overall and per patient, the type of mobilization activities completed, location (ICU, HDU or neurosurgical ward), whether the patient was mobilized with an EVD, tracheostomy or endotracheal tube in situ, equipment used and number of staff required for each mobilization session. Vasopressor use during mobilization was documented including whether dosage was increased and if target blood pressure set by the medical team was maintained.

Each patient was assessed by a physical therapist to determine their suitability to engage in early mobilization. For patients that did not proceed to mobilization, the type of reported barriers to mobilization was recorded in the patient’s medical file. Safety events were recorded by the treating physical therapist and were collected over 14 days or until discharge (whichever occurred first). A safety event was defined as an event that led to early cessation of the mobilization session with safety criteria outlined in the supplementary material (Suppl. Material 2). Serious adverse events were defined as a fall, cardiac arrests or arrhythmias, line removal (non-invasive or invasive) or unplanned extubation.

To compare mobilization outcomes between aSAH patients with a good clinical status and poor clinical status on admission, time to out-of-bed mobilization, proportion of patients that achieved independent out-of-bed mobilization milestones, highest level of mobilization achieved during the first 14 days post-repair and day achieved, maximum level of mobilization achieved at day 14 using the MSAS, number of mobilization sessions completed per patient, total number of patients that did not mobilize and frequency of reported barriers to mobilization were collected by research staff.

### Statistical analysis

Statistical analysis was performed using statistical software package, R (R version 4.3.2; R Core Team; Vienna, Austria). To describe current mobilization practices (including mobilization frequency and highest mobility level achieved), reported barriers and safety concerns, continuous variables were summarized as mean (standard deviation) for parametric data or median (interquartile range) for nonparametric data and categorical variables were presented as frequency counts and percentages.

Patients were dichotomized into 2 independent groups based on the initial WFNS scale (classified as “good” when WFNS grade ≤ 2 or “poor” when WFNS grade 3 to 5); mobilization variables (including time to out-of-bed mobilization, proportion of patients that achieved independent out-of-bed mobilization milestones, highest level of mobilization achieved by Day 14 and frequency of barriers to mobilization) were compared between groups using the Wilcoxon rank sum test for continuous data and using Pearson chi-square test or Fisher exact test for categorical data presented as percentages and frequencies. Missing data was not imputed for 14-day mobility and functional outcome data, where patients died within 14 days from baseline.

A sample size calculation for a single proportion assumed an 85% recovery of independent walking^24^ and a width of the 95% confidence interval of 14 (78 to 92), generating an estimate of 100 evaluable participants.

## Results

### Patient characteristics

**Figure 1:**
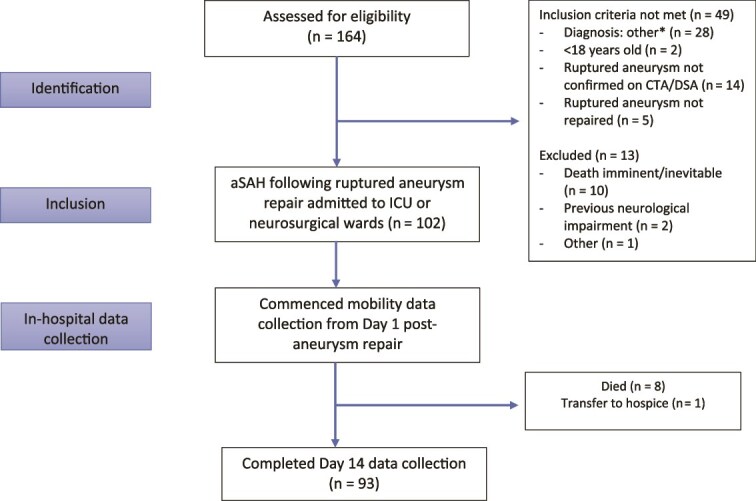
Flow of Participants through Study. Diagnosis: Other* - Patients Screened Due to Suspected aSAH on Admission. Abbreviations: aSAH =  aneurysmal subarachnoid hemorrhage; CTA = computed tomography angiography; DSA = digital subtraction angiography; ICU = intensive care unit.

A total of 115 patients met the inclusion criteria with 13 excluded (Figure 1). Of the 102 patients recruited, 93 patients survived to day 14. There were no deaths associated with early mobilization in the patients that died. Demographic, clinical characteristics and medical and surgical interventions are summarized in Table 1. The mean (SD) age was 56.7 (12.3) years and 67.6% of patients were female. Aneurysm rupture occurred during an elective procedure to endovascularly repair an unruptured aneurysm procedure in 2 patients and hence clinical severity could not be graded using the WFNS grading scale. Based on the WFNS scale (*n* = 100), 69 (69.0%) and 31 (31.0%) classified as good and poor respectively.

**Table 1:** Demographic and hospital data*^a^*

**Variable**	**N = 102**
**Patient demographic data**	
Age (y), mean (SD)	56.7 (12.3)
Sex, female n (%)	69 (67.6)
Co-morbidity, n (%)	
Hypertension	33 (32.4)
Diabetes (type 1 or type 2)	6 (5.9)
Cardiac other*^b^*	4 (3.9)
Respiratory*^c^*	9 (8.8)
Anxiety and/or depression	10 (9.8)
Premorbid modified Rankin Scale, n (%)	
0	96 (94.1)
1	2 (2.0)
2	2 (2.0)
3	2 (2.0)
**aSAH severity scores and characteristics**	
WFNS grade on admission, n (%)	
I	51 (50.0)
II	18 (17.6)
III	4 (3.9)
IV	9 (8.8)
V	18 (17.6)
Other: aneurysm rupture during elective procedure	2 (2.0)
Aneurysm location	
MCA branch	25 (24.5)
ACA branch	44 (43.1)
ICA branch	8 (7.8)
PCA branch	23 (22.5)
Indeterminate	2 (2.0)
Modified Fisher Scale, n (%)	
0	3 (2.9)
1	12 (11.8)
2	13 (12.7)
3	23 (22.5)
4	51 (50.0)
Admitted to ICU at baseline*^d^*, n (%)	46 (45.1)
**Surgical and medical interventions**	
Time from ictus to securing the aneurysm (d), median (IQR)	0.9 (0.5, 2.3)
Endovascular repair via coiling, n (%)	57 (55.9)
Surgical clipping via craniotomy, n (%)	48 (47.1)
External ventricular drain, n (%)	52 (51.0)
Mechanically ventilated at baseline*^d^*, n (%)	39 (38.2)
Tracheostomy inserted, n (%)	2 (2.0)
Active treatments for delayed cerebral ischemia, n (%)	
Intravenous vasopressors	24 (23.5)
Balloon angioplasty	12 (11.8)
Intraarterial vasodilators	20 (19.6)

^a^ Abbreviations: ACA = anterior cerebral artery; aSAH = aneurysmal subarachnoid hemorrhage; ICA = internal carotid artery; ICU = intensive care unit; IQR = interquartile range; MCA = middle cerebral artery; N = total sample size; n = total number of patients; PCA = posterior cerebral artery; SD = standard deviation; WFNS = World Federation of Neurological Surgeons.

^b^ Cardiac other = angina, congestive heart failure, heart disease, myocardial infarction.

^c^ Respiratory = asthma, chronic obstructive pulmonary disease, acute respiratory distress syndrome, emphysema.

^d^ Within 72 hours of hospital admission.

Overall 42 (41.2%) participants experienced at least 1 of the following neuroradiological complications: delayed cerebral ischemia (*n* = 30, 29.4%), radiological vasospasm (*n* = 39, 38.2%) and cerebral infarction identified on imaging (*n* = 18, 17.6%). Active treatment for delayed cerebral ischemia, which included intravenous vasopressors, intra-arterial vasodilators or balloon angioplasty, occurred in 29 (28.4%) of patients. The median length of stay in hospital was 14.8 (IQR = 10.9 to 22.3) days and for those admitted to ICU during their hospital stay (55.9%), their median ICU length of stay was of 8.8 (IQR = 3.4 to 13.8) days. There were 44 (43.1%) patients who required mechanical ventilation during their hospital admission with an average total median (IQR) duration of 6.5 (2.1 to 9.0) days. Pulmonary embolism occurred in 5 (4.9%) patients and deep vein thrombosis in 1 (1.0%) patient. Of the 102 patients included in the study, 55 (53.9%) returned to their previous living situation from hospital, 23 (22.5%) were transferred to another acute hospital, 12 (11.8%) died in hospital, 10 (9.8%) were discharged to rehabilitation and 2 (2.0%) were discharged to a hospice.

### Mobilization description

Ninety (88.2%) of the 102 patients received mobilization with physical therapy during the first 14 days post-repair, with descriptions of mobilization outcomes outlined in Table 2. The highest level of mobilization measured using the MSAS was achieved at a median (IQR) of 6 days (2.0 to 10.0) post-aneurysm repair. Patients who mobilized with physical therapy engaged in a median (IQR) of 4.0 (2.0 to 6.0) sessions during the 14 days following repair.

**Table 2:** Mobilization outcomes overall during the first 14 days post-aneurysm repair and when dichotomized by clinical status on admission*^a^*

**Mobilization data**	All patients N = 102	Good clinical status WFNS 1,2 n = 69	Poor clinical status WFNS 3–5 n = 31	*P* of difference between grades
Time from aneurysm repair to out-of-bed mobilization milestones (d), median (IQR):				
First sit on edge of bed*^b^*	1.0 (0.0–3.0)	1.0 (0.0–2.0)	3.0 (1.0–7.0)	.002
First sit to stand*^c^*	1.0 (0.0–2.0)	1.0 (0.0–2.0)	3.0 (0.0–8.0)	.029
First walk 10m*^d^*	1.0 (0.0–2.3)	1.0 (0.0–2.0)	3.0 (2.3–7.5)	.002
Number (%) of patients that achieved independent out-of-bed mobilization milestones by Day 14				
Independent sitting*^e^*	57 (55.9)	51 (73.9)	5 (16.1)	<.001
Independent standing*^f^*	50 (49.0)	46 (66.7)	3 (9.7)	<.001
Independent walking*^g^*	50 (49.0)	45 (65.2)	4 (12.9)	<.001
Highest level of mobilization achieved MSAS during first 14 days, median score (IQR)	32 (10–36)	36 (30–36)	10 (6–20)	<.001
Day highest level of mobilization achieved post-aneurysm repair, median score (IQR)	6.0 (2.0–10.0)	6.0 (2.0–10.0)	4.0 (0.5–10.5)	.413
Total patients that did not mobilize during first 14 days, n (%)	12 (11.8)	4 (5.8)	8 (25.8)	.01
MSAS score at day 14, median (IQR)*^h^*	32 (15–36) Missing = 9	36 (30–36) Missing = 4	10 (7–24) Missing = 5	<.001

^a^ Abbreviations: IQR = interquartile range; m = metres; MSAS = Mobility Scale for Acute Stroke; N = total sample size; n = total number of patients; WFNS = World Federation of Neurological Surgeons.

^b^ MSAS item 2: score 2–6.

^c^ MSAS item 4, score 2–6.

^d^ MSAS item 6: any score 2–6 (ie, first walk 10 metres with or without gait aid and with or without assistance).

^e^ Achieved score of 6 on MSAS item 2.

^f^ Achieved score of 6 on MSAS item 4.

^g^ Achieved score of 6 on MSAS item 6.

^h^ MSAS at day 14 were calculated based on patients who survived up to 14-day data collection.

There were 603 mobilization sessions attempted; 410 (68.0%) sessions occurred with physical therapy during the first 14 days post-repair. The location, number of staff required and type of mobilization activities are outlined in Table 3. One-third of mobilization sessions occurred with an EVD (*n* = 126, 30.7%) in place. It was uncommon for patients to be mobilized with a tracheostomy (*n* = 4, 1.0%) or endotracheal tube (*n* = 4, 1.0%). The majority of sessions (*n* = 330, 80.5%) did not require any equipment for mobilization activities. When equipment was used during a mobilization session, a hoist machine was most commonly used (*n* = 51, 12.4%). The use of walking frames (*n* = 8, 2.0%) and standing transfer assist devices (*n* = 6, 1.5%) to mobilize were rare. The type of equipment used during the 410 mobilization sessions is outlined in the electronic supplementary material (Suppl. Material 3).

**Table 3:** Mobilization sessions completed with physical therapist during the first 14 days post-aneurysm repair*^a^*

**Mobilization session data**	N = 410
Location of mobilization session, n (%)	
ICU	66 (16.1)
HDU	314 (76.6)
Neurosurgical ward	30 (7.3)
Number of staff required for mobility session, n (%)	
1	219 (53.4)
2	156 (38.0)
3	31 (7.6)
4	4 (1.0)
Types of exercise achieved during mobility sessions*^b^*, n (%)	
Active bed mobility	79 (19.3)
Sitting balance	93 (22.7)
Sit to stand practice	101 (24.6)
Standing balance	72 (17.6)
Tilt table	2 (0.5)
Step transfer	62 (15.1)
Walking	269 (65.6)
Stairs practice	37 (9.0)
ADL training	7 (1.7)

^a^ Abbreviations: ADL = activities of daily living; HDU = high dependency unit; ICU = intensive care unit; N = Total number of mobilization sessions; n = number of mobilization sessions.

^b^ Each mobility session may have included more than 1 type of exercise.

### Barriers to mobilization

Physical therapists indicated a barrier to mobilization in 193 (32.0%) sessions (Table 4). The most common barriers were patient drowsiness (*n* = 48, 24.9%), hemodynamic instability (*n* = 43, 22.3%), mechanical ventilation (*n* = 35, 18.1%), patient refusal (*n* = 24, 12.4%), and patient sedation (*n* = 20, 10.4%). Supplementary Material 4 illustrates the number of sessions per day during the first 15 days post-aneurysm repair in which at least 1 barrier to mobilization occurred.

**Table 4:** Reported barriers to mobilization

**Barrier type** ^***a***^	**N = 193** ^***b***^
Neurological instability*^c^*	80 (41.5)
Hemodynamic instability	43 (22.3)
Mechanically ventilated	35 (18.1)
Patient refusal	24 (12.4)
Sedation	20 (10.4)
Confused and not following commands	17 (8.8)
Other	14 (7.3)
Agitation or delirium	14 (7.3)
Organizational barriers*^d^*	10 (5.2)
Communication barriers*^e^*	9 (4.7)
Pain	6 (3.1)
Respiratory instability	5 (2.6)
Nausea or vomiting	3 (1.6)

a Physical therapists reported 1 or more barrier types for each mobilization session.

b n (%).

c Neurological instability = active treatment for high intracranial pressures (ICPs), recent seizures, active deterioration in neurology, external ventricular drain (EVD) not able to clamped for mobility, excessive drowsiness/comatosed, recent/awaiting neurological procedure.

d Organizational barriers = equipment required for mobilization not available, limited staffing (numbers and expertise), time constraints, awaiting helmet (if required), impending ward transfer from intensive care unit, impending discharge form hospital.

e Communication barriers = Clearance to mobilize not obtained or documented from senior medical staff, poor timing of reducing sedation, unable to proceed secondary to activities of daily living being undertaken, timing of procedures.

### Safety events during mobilization

In 34 of 410 (8.3%) mobilization sessions at least 1 safety event was reported. Increased headache (*n* = 6, 1.5%), hypotension (*n* = 6, 1.5%), light-headedness (*n* = 6, 1.5%) and reduced level of consciousness (*n* = 5, 1.2%) were the most frequently reported safety events. All reported safety events are provided in the supplementary material (Suppl. Material 5). Of these safety events, 13 (38.2%) occurred in the ICU and 21 (61.8%) in the HDU. No invasively inserted lines were removed during physical therapist sessions. Additionally, there were no medical emergency team calls, cardiac arrest or cardiac arrythmias, falls or new focal neurology that occurred during any of the mobilization sessions. Of the 22 (5.4%) sessions with intravenous vasopressors in situ, 8 sessions (36.4%) required an increase of vasopressor dose during the session and on one of these occasions the target blood pressure set by the medical team was not maintained.

### Association between clinical status and mobilization activities

**Figure 2:**
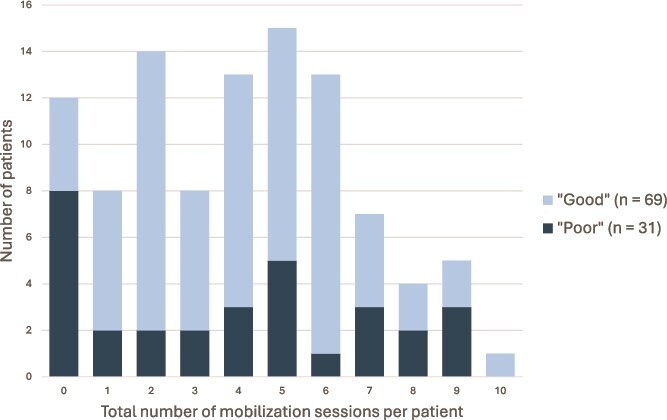
Total Number of Mobilization Sessions Completed per Patient when Dichotomized by Clinical Severity (ie, WFNS Good and Poor Clinical Status Categorization) (N = 100).

Patients admitted with good clinical status were significantly more likely to progress to independent out-of-bed mobilization within 2 weeks compared with those admitted with poor clinical status. By 2 weeks, independence in walking was achieved in 65.2% of patients with good clinical status and 12.9% of those with poor clinical status (*P* < .001). Detailed results are outlined in Table 2. The total number of sessions completed per patient over the first 14 days post-aneurysm repair according to good or poor clinical status is illustrated in Figure 2. A median (IQR) of 1.0 (0.0 to 3.0) session did not occur due to reported barriers for those classified as “good” compared with a median (IQR) of 2.0 (1.5 to 3.5) sessions in those classified as “poor” (*P* = .005).

## Discussion

In this prospective study, a median of 4 mobilization sessions were completed with physical therapists during the early acute phase (ie, within the first 14 days after aneurysm repair). A total of 88% of patients mobilized early following aneurysm repair. One-third of planned mobilization sessions did not commence due to 1 or more reported barriers with neurological instability (41.5%), hemodynamic instability (22.3%) and mechanical ventilation (18.1%) most commonly hindering mobilization. Although safety concerns during mobilization occurred in 8.3% of sessions, no serious adverse events occurred. Patients with a good clinical status on admission were more likely to mobilize to standing early and achieve independence across all mobility items measured by the MSAS compared with those who were admitted with a poor clinical status.

Recent clinical practice guidelines advocate for early rehabilitation post-aneurysm repair to enhance functional outcomes and reduce hospital length of stay.^8^^,^^11^ However, unlike the specific recommendations outlined in the stroke guidelines for ischemic stroke,^8^^,^^25^ there are no standardized protocols for aSAH patients, with variability in timing, dose and type of mobilization activities based on studies investigating early mobilization programs.^26–30^

A dose–response analysis of the A Very Early Rehabilitation (AVERT) trial, a multi-center, RCT of 2104 patients, found that frequent out-of-bed mobilization sessions each day during the first 2 weeks in the acute setting was associated with improved functional outcomes at 3 months following ischemic and intracerebral stroke.^31^ Our study described lower mobilization frequency, with a median of 4 mobilization sessions completed with physical therapists within the first 14 days. These differences may be attributable to several factors including post-bleed headaches, the requirement for external ventricular drains and mechanical ventilation and close monitoring for neurological complications that are more frequently seen in patients with aSAH. Notably almost half of sessions required 2 or more staff members to assist with mobilization activities, which may reflect the complexity associated with mobilizing patients with aSAH. Despite these potential challenges, walking was by far the most common type of mobilization activity undertaken by patients during the first 2 weeks. This tendency to engage in higher levels of mobilization (ie, walking activities), may have, in part, contributed to the low incidence of pulmonary embolism and deep vein thrombosis during hospitalization. Future research should focus on defining optimal delivery of early rehabilitation, including dose and content, to enhance functional outcomes whilst carefully considering the impact of post-bleed symptoms and level of impairment.

Despite two-thirds of the patients in our cohort demonstrating no neurological deficits on admission (WFNS grade 1 or 2), over half of patients required admission to ICU and neurological instability frequently prevented mobilization. Of note, there were 12 (11.8%) of patients who never engaged in mobilization activities, including 4 patients classified as WFNS grade 1 or 2. This highlights that even for those predicted to have a favorable prognosis, medical instability and neurological complications impacting on mobilization were relatively common. Since fifty percent of the cohort were classified as grade 4 on the modified Fisher scale, the amount of blood on imaging may have served as a more important predictor of a patient's potential to engage in mobilization activities during hospitalization. However, the benefits versus harm of mobilization activities during the high-risk period for neuroradiological complications remains unclear. One previous study reported early mobilization in aSAH was associated with harm.^32^ This single-center randomized clinical trial of 65 patients compared early verticalization commencing on day 2 to 5 compared with verticalization on day 12 and reported a significantly higher proportion of patients with cerebral ischemia on CT (*P* = .04) and hemiplegia (*P* = .035) at discharge in the early mobilization group. Conversely, there have been several observational studies reporting that mobilization did not reduce cerebral blood flow^33^^,^^34^ and had potential to reduce symptomatic vasospasm in hospital.^16^^,^^35^^,^^36^ With lack of randomized controlled trials and heterogeneity of mobilization programs in published studies, the effect of early mobilization requires further investigation.

Historically there have been concerns regarding the safety of out of bed mobilization with patients with aSAH, including aggravation of neurological complications, supporting prescription of strict bedrest post-bleed. Specifically, recent surveys of neurosurgeons and physical therapists in Europe, Australia, and New Zealand have found that concerns regarding mobilization post-aSAH were common in relation to EVD dislodgement, reduced cerebral perfusion and hemodynamic instability during mobilization activities.^37–39^ Despite this, observational studies have reported early mobilization to be safe.^16^^,^^17^^,^^24^^,^^26^^,^^28^^,^^40^^,^^41^ A systematic review meta-analysis of 4 studies reported a cumulative incidence of 6% (24 out of 900 mobilization sessions) for adverse events related to early mobilization where the most common type of safety event reported was hemodynamic instability (*n* = 21, 2.3%).^42^ No falls were reported in any of these studies. Where a meta-analysis could not be conducted as part of this systematic review, there were 4 serious adverse events reported where invasive line removal occurred. Interestingly, the present study found a slightly higher incidence of adverse events with 8.3% of mobilization sessions ceasing early due to safety concerns. Of note, there was no dislodgement of lines that were reported during mobilization despite one-third of patients mobilizing with an EVD in place. This is similarly reported in studies examining the safety of mobilization practices in patients with EVDs where adverse events ranged from 0.8% to 5.9%,^40^^,^^41^ without unintentional removal of lines during mobility. In the current study while safety events were not infrequent (8.3%), no safety events occurred that led to prolonged hospital admission.

### Strengths and limitations

The strengths to this study include that there was no loss to follow-up. We provide fully completed mobilization data from physical therapist sessions until day 14 or discharge for those who survived and prospective data collection was achieved. Limitations include the lack of generalizability from a study of a single-site and that descriptive observational data can only provide associations, not explore causation. Although the number of early mobilization sessions delivered was collected, data on the intensity and duration of mobilization sessions were not recorded which may limit the understanding of the potential impact of overall mobilization dose on mobilization outcomes. This is an important area for future research. Mobilization data were collected by physical therapists only, and mobilization activities that occurred with other staff (such as nursing staff), or outside of dedicated therapy time were not included.

## Conclusion

This study highlights the complexities involved with mobilizing patients following aSAH. Whilst most enrolled patients engaged in early mobilization with physical therapists, only half of patients achieved independent walking by 2 weeks. Reported safety events were most commonly due to post-bleed symptoms, hypotension or reduced consciousness; however, there were no serious adverse events related to early mobilization. Physiological instability, including neurological and hemodynamic issues, were common barriers to mobilization. In summary, study findings emphasize the need for tailored mobilization strategies based on a patient’s post-bleed symptoms and physiological status. Further research to explore the effects of timing, dosage and content of mobilization programs on physical recovery during the acute hospital stay is warranted.

## Supplementary Material

PTJ-2025-0242_R2_aSAH_descriptive_study_ESupplementary_v3_25032026_pzag031

## Data Availability

The datasets generated during and/or analyzed during the current study are available from the corresponding author on reasonable request.
