# Supplementary material for: Early mobilization in patients with aneurysmal subarachnoid hemorrhage: a prospective observational study
Source: Phys Ther. 2026 Mar 27;106(4):pzag031. doi: 10.1093/ptj/pzag031 (PMC13134040; doi:10.1093/ptj/pzag031)
Supplement: PTJ-2025-0242_R2_aSAH_descriptive_study_ESupplementary_v3_25032026_pzag031 [file ptj-2025-0242_r2_asah_descriptive_study_esupplementary_v3_25032026_pzag031.pdf]

Electronic Supplementary Material

Supplementary Material 1. Case Report Forms – Demographic, baseline and mobility data

In hospital data collection - Case report form 1	Source
**Demographic and baseline data**<br>• Age<br>• Gender<br>• Co-morbidities<br>• Pre-admission level of function (mRS[13])	 EMR<br>EMR<br>EMR<br>EMR or if unavailable, the score was assigned by the study researcher using documentation of functional status and independence recorded in the electronic medical record.
**Illness severity Data**<br>• GCS on admission to ED<br>• WFNS[14] grade on admission<br>• modified Fisher grade[18] on admission	 EMR<br>EMR or Neurosurgeon*<br><br>EMR or Neurosurgeon*<br><br>*Neurosurgeon (study investigator) graded on CT or MRI imaging retrospectively where not documented in EMR
**Admission data**<br>• Date/time of hospital admission and discharge<br>• Time from SAH to repair of ruptured aneurysm	 EMR<br>EMR
**ICU data**<br>• Day post-admission admitted/ discharge to ICU<br>• Mechanical ventilation (Yes/No)<br>o Start and stop time of mechanical ventilatory support<br>• Tracheostomy (Yes/No)<br>• ICU length of stay	 EMR<br>EMR<br><br><br>EMR<br>EMR
**Neurosurgical intervention performed**<br>• Endovascular repair (coiling or stent)<br>• Surgical clipping<br>• EVD insertion<br>• Interventions for vasospasm<br>o Vasopressors<br>o Intra-arterial vasodilators<br>o Intra-arterial angioplasty	 EMR<br>EMR<br>EMR<br>EMR
**Complications**<br>• Radiological vasospasm detected on imaging or DSA<br>• Cerebral infarction detected on CT/MRI	 EMR<br><br>EMR or Neurosurgeon*

• Delayed cerebral ischaemia<br>• Pulmonary embolism<br>• Deep vein thrombosis	EMR or Neurosurgeon*<br>EMR<br>EMR<br><br>*Neurosurgeon (study investigator) graded based on CT or MRI imaging retrospectively where not documented in EMR
**Outcome data**<br>• In-hospital mortality<br>• Discharge destination from acute hospital	 EMR<br>EMR
**Daily mobilisation data – Case report form 2**	**Source**
• Date and time of mobilization<br>• Location (ICU/HDU /Ward)<br>• Barrier to mobilization<br>• EVD present (yes / no)<br>• Tracheostomy present (yes / no)<br>• Vasopressor infusion (yes / no)<br>• Vasopressor dose increased during session (yes / no)<br>• Types of exercises completed<br>• Number of staff required<br>• Equipment used<br>• MSAS[21] score<br>• Type of safety concern that led to early cessation of the session	Data from routine physical therapy sessions conducted within 14 days of aneurysm repair were documented in the electronic medical record by the treating physiotherapist and subsequently extracted by a study researcher.
**14-day Outcome data – Case report form 3**	**Source**
• MSAS[21] score at 14 days	Measured and recorded in EMR by the treating physical therapist within 24-48 hours of day 14 post-aneurysm repair or discharge (whichever soonest). The study researcher subsequently extracted this data from electronic medical records.

Legend**:** mRS = modified Rankin Scale, EMR = electronic medical records, GCS = Glasgow Coma Scale, ED = Emergency Department, WFNS = World Federation of Neurological Surgeons scale, CT = computed tomography, MRI = Magnetic resonance imaging, SAH = subarachnoid haemorrhage, ICU = intensive care unit, EVD = external ventricular drain, DSA = digital subtraction angiography, HDU = high dependency unit, MSAS = Mobility Scale for Acute Stroke.

Supplementary Material 2. Description of Safety Criteria for Early Cessation of Mobilization

Concerns over neurological status	▪ Reduced level of consciousness<br>▪ Increased headache<br>▪ New onset confusion<br>▪ New focal neurological deficit<br>▪ Seizures<br>▪ Increase in ICP≥ 20mmHg<br>▪ Acute deterioration in neurology
Concerns over cardiovascular status	▪ Bradycardia <40bpm or tachycardia >130 bpm<br>▪ Hypotension SBP <90 or MAP <70 or outside of target limits set by neurosurgeons or intensivist<br>▪ Hypertension SBP >200mmHg or MAP > 110mmHg or outside of target limits set by neurosurgeon or intensivist<br>▪ Acute chest pain<br>▪ Cardiac arrythmia<br>▪ Cardiac arrest
Concerns over respiratory status	▪ Sustained desaturation of percutaneous pulse oximetry < 88% > 2 minutes<br>▪ Respiratory rate < 5 or > 40 breaths/minute<br>▪ Signs of respiratory distress or unacceptable breathing pattern
Patient-related factors	▪ Patient appears distressed<br>▪ Patient reporting light-headedness
Removal of lines	▪ Line removal requiring urgent replacement<br>▪ Line removal not requiring immediate replacing
Other	▪ Falling to the floor<br>▪ Medical emergency team call during mobilization<br>▪ Other

bpm = beats per minute, ICP = intracranial pressure, MAP = mean arterial pressure, SBP = systolic blood pressure,

Supplementary Material 3. Total number of equipment required during mobilization sessions completed with physical therapy

Equipment required	n/N (%)
- Single point stick	0/410 (0.0)
- Walking frame (2WW/4WW)	5/410 (1.2)
- Gutter frame	3/410 (0.7)
- Hoist machine	51/410 (12.5)
- Standing machine	0/410 (0.0)
- Sara Stedy	6/410 (1.5)
- Tilt table	4/410 (1.0)
- Hand or leg weights	0/410 (0.0)
- Cycle ergometer	0/410 (0.0)
- Electrical stimulation	0/410 (0.0)
- Sara Combilizer	2/410 0.5)
- Standard chair	13/410 (3.2)
- Tilt-in-space wheelchair	19/410 (4.7)
- Chair accessed via slide transfer	0/410 (0.0)
- Patslide	8/410 (2.0)
- Other	7/410 (1.7)

*mobility sessions may have used >1 type of equipment
n = number of mobilization sessions where equipment required, N = total number of mobilization sessions completed with physical therapy, 2WW = two wheel walker, 4WW = four wheel walker

Supplementary Material 4. Total number of mobilization sessions that did not commence due to one or more barriers (n=193)

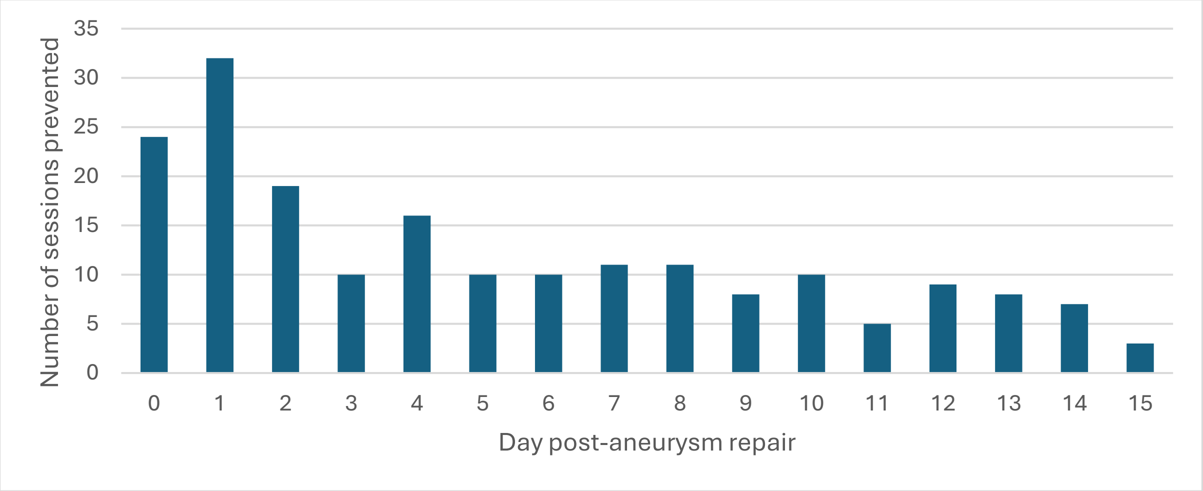

* More than one barrier type may have been reported for each mobilization session

Supplementary Material 5: Total number (%) of safety events reported by physical therapists that led to early cessation of mobilisation

Safety category	Safety event type	n	% of N
Fall	Falling to the floor	0	0.0
Concerns over neurological status	Reduced level of consciousness	5	1.2
	Increased headache	6	1.5
	New onset confusion	0	0.0
	New focal neurological deficit	0	0.0
	Seizures	0	0.0
	Increase in ICP≥ 20mmHg	1	0.2
	Acute deterioration in neurology	0	0.0
Concerns over cardiovascular status	Bradycardia <40bpm or tachycardia >130 bpm	2	0.5
	Hypotension SBP <90 or MAP <70 or outside of target limits set by neurosurgeons or intensivist	6	1.5
	Hypertension SBP >200mmHg or MAP > 110mmHg or outside of target limits set by neurosurgeon or intensivist	1	0.2
	Acute chest pain	0	0.0
	Cardiac arrythmia	0	0.0
	Cardiac arrest	0	0.0
Concerns over respiratory status	Sustained desaturation of percutaneous pulse oximetry < 88% > 2 minutes	0	0.0
	Respiratory rate < 5 or > 40 breaths/min	0	0.0
	Signs of respiratory distress or unacceptable breathing pattern	0	0.0
Lines	Line removal requiring urgent replacement	0	0.0
	Line removal not requiring immediate replacing	0	0.0
Physiological other	Patient appears distressed	3	0.7
	MET call during mobilisation	0	0.0
	Patient reporting light-headedness	6	1.5
Other concerns	Impulsive and/or distractible	2	0.5
	Restless and/or agitated	2	0.5
	Knee giving way	2	0.5
	Near fall to floor from edge of bed	1	0.2
Total		37*	

bpm = beats per minute, ICP = intracranial pressure, MAP = mean arterial pressure, MET = Medical emergency team, n = total number of safety events, N = 410 mobilization sessions, SBP = systolic blood pressure,

*Physical therapists may have reported greater than 1 safety concern for the mobilisation session

References:

13. van Swieten JC, Koudstaal PJ, Visser MC, Schouten HJ, van Gijn J. Interobserver agreement for the assessment of handicap in stroke patients. *Stroke*. 1988;19(5):604-607. doi:10.1161/01.STR.19.5.604

14. Rosen DS, Macdonald RL. Subarachnoid hemorrhage grading scales: a systematic review. *Neurocrit Care*. 2005;2:110-118.

18. Frontera JA, Claassen J, Schmidt JM, et al. Prediction of symptomatic vasospasm after subarachnoid hemorrhage: the modified fisher scale. *Neurosurgery*. 2006;59(1):21-27.

21. Simondson J, Goldie P, Brock K, Nosworthy J. The mobility scale for acute stroke patients: intra-rater and inter-rater reliability. *Clin Rehabil*. 1996;10(4):295-300.
